# Editorial: The ubiquitin-proteasome system and cellular signaling: mechanisms and regulatory roles in cancer and infectious diseases

**DOI:** 10.3389/fcell.2026.1812881

**Published:** 2026-02-25

**Authors:** Sehbanul Islam, Aabid Hussain, Aftab Alam, Mohammad Owais Ansari, Haris Saeed, Sajid Khan

**Affiliations:** 1 Cancer Biology Division, Perelman School of Medicine, University of Pennsylvania, Philadelphia, PA, United States; 2 Genomic Sciences and Systems Biology, Cleveland Clinic Research, Cleveland Clinic, Cleveland, OH, United States; 3 Department of Immunology, Roswell Park Comprehensive Cancer Center, Buffalo, NY, United States; 4 Department of Pharmacology and Regenerative Medicine, University of Illinois Chicago, Chicago, IL, United States; 5 Department of Medicine, Keck School of Medicine, University of Southern California, Los Angeles, CA, United States; 6 Department of Biochemistry and Structural Biology, The University of Texas San Antonio, UT Health Science Center, San Antonio, TX, United States

**Keywords:** apotosis, cancer, E3 ubiquitin ligase, proteolysis-targeting chimeric molecule (PROTAC), ubiquitin proteasome system

The ubiquitin–proteasome system (UPS) is a central mechanism for maintaining cellular homeostasis through the regulated turnover of proteins (Pickart, 2001; Hershko and Ciechanover, 1998). Ubiquitination occurs through a cascade of three enzymes: an E1 ubiquitin-activating enzyme, an E2 ubiquitin-conjugating enzyme, and an E3 ubiquitin ligase. In this process, ubiquitin is first activated by the E1 enzyme in an ATP-dependent manner and subsequently transferred to an E2 conjugating enzyme. E3 ubiquitin ligases then confer substrate specificity by catalyzing the transfer of ubiquitin from the E2-ubiquitin conjugate to lysine residues on target proteins (Yang et al., 2021). During ubiquitination, ubiquitin molecules are sequentially linked through one of ubiquitin’s seven lysine residues to form polyubiquitin chains. Notably, lysine-11– and lysine-48–linked chains act as canonical signals for recognition and degradation by the 26S proteasome (Islam et al., 2021a).

E3 ubiquitin ligases are broadly classified into four major families based on their structural domains and mechanisms of action: RING (Really Interesting New Gene), HECT (Homology to E6AP C-Terminus), U-box, and RBR (RING-between-RING) (Yang et al., 2021). RING E3 ligases mediate the direct transfer of ubiquitin from the E2 enzyme to the substrate. In contrast, HECT ligases form a transient thioester intermediate with ubiquitin before transferring it to the target protein. Among these families, RING E3 ligases are the largest and most diverse, existing as monomeric, dimeric, or multi-subunit complexes, many of which are assembled around Cullin scaffold proteins (Mishra et al., 2025).

By regulating protein stability, E3 ligases control a wide range of essential cellular processes, including cell cycle progression, proliferation, differentiation, autophagy, DNA damage responses, apoptosis, and signal transduction (Welcker et al., 2008; Agrawal et al., 2022; Islam et al., 2021b; Islam et al., 2021c; Paul et al., 2019). Notably, they modulate key oncogenic signaling pathways, including the PI3K/AKT, β-catenin, and NF-κB pathways, which are critical for cancer cell growth and survival (Paul et al., 2019; Zhan et al., 2022). Consistent with these roles, dysregulation of E3 ligase expression or activity is a hallmark of many cancers and directly contributes to tumorigenesis (Dagar et al., 2023; Liu et al., 2024; Islam et al., 2022).

The clinical success of proteasome inhibitors, such as bortezomib, provided the first validation of the UPS as a therapeutic target (Lub et al., 2016). Beyond conventional small-molecule inhibition, the UPS can be hijacked to achieve the selective elimination of oncogenic proteins through targeted protein degradation (TPD) strategies, including proteolysis-targeting chimeras (PROTACs) and molecular glue degraders (Hu et al., 2024; Barghout, 2021). These approaches have transformed the cancer therapeutic landscape by allowing the targeting of previously “undruggable” proteins, such as transcription factors and scaffold proteins (Kelm et al., 2023; Liu et al., 2020; Saeed et al., 2023; Kim et al., 2023).

This editorial highlights two original research articles and two review articles that collectively illustrate recent advances and emerging challenges in UPS- and TPD-based cancer therapeutics. In a research article, Cen et al. demonstrated that elongation of very-long-chain fatty acid protein 2 (ELOVL2)-mediated stabilization of the androgen receptor (AR) contributes to enzalutamide resistance in prostate cancer. Enzalutamide, a second-generation AR inhibitor, is a cornerstone therapy for castration-resistant prostate cancer (CRPC); however, the emergence of drug resistance remains a major clinical challenge. Through bioinformatic analyses and subsequent experimental validation, the authors identified significant upregulation of ELOVL2 in enzalutamide-resistant prostate cancer cells. Furthermore, functional analysis studies revealed that depletion of ELOVL2 markedly suppressed tumor cell proliferation and restored sensitivity to enzalutamide in resistant models. Mechanistically, ELOVL2 promotes enzalutamide resistance by stabilizing AR through inhibition of ubiquitin–proteasome–mediated degradation. However, the mechanism by which ELOVL2 interfaces with the UPS remains to be elucidated. Collectively, these findings identify ELOVL2 as a critical regulator of AR stability, suggesting that targeting ELOVL2 represents a promising therapeutic strategy to overcome enzalutamide resistance.

The second article by Li et al. examines how mebendazole affects the ubiquitination and stability of the tumor necrosis factor receptor 1 (TNF1)-associated death domain protein (TRADD) in non-small cell lung cancer (NSCLC). TRADD is known to trigger pro-apoptotic autophagy, but the E3 ubiquitin ligase responsible for its ubiquitination has not been well defined. Using *in silico* prediction and biochemical assays, the authors identified PELI3 as a candidate E3 ubiquitin ligase that binds to and ubiquitinates TRADD, leading to its proteasome-mediated degradation in NSCLC cells. Overexpression of PELI3 decreases TRADD protein levels without affecting its mRNA, shortens TRADD half-life, and increases polyubiquitination, whereas PELI3 knockdown stabilizes TRADD. Treatment with mebendazole reduces PELI3 expression and increases TRADD protein levels, resulting in a dose-dependent decrease in NSCLC cell viability. These findings support a model in which mebendazole inhibits PELI3-mediated ubiquitination of TRADD, stabilizing TRADD and implicating this axis as a potential therapeutic target in NSCLC.

The review article by Chen et al. presents an in-depth overview of the involvement of E3 ubiquitin ligases in the pathogenesis and treatment of multiple myeloma (MM). The authors systematically summarized how distinct E3 ligases regulate MM pathogenesis through multiple mechanistic axes, including degradation of oncoproteins, modulation of signaling pathway components, control of cell cycle regulators, regulation of apoptosis-related proteins, maintenance of DNA damage response factors, and governance of autophagy-related proteins. Importantly, the review highlights emerging E3 ligase-based therapeutic strategies, such as molecular glue degraders, exemplified by the clinically approved immunomodulatory drugs, alongside PROTACs and other small-molecule inhibitors, underscoring their translational relevance for MM therapy.

Lastly, the mini-review by Li et al. focuses on the E3 ubiquitin ligase TRIM21, a member of the TRIM family with RING-domain E3 ligase activity, and its complex roles in cancer biology. The authors describe the multidomain structure of TRIM21 and its regulation at both transcriptional and post-translational levels. TRIM21’s activity has been implicated in diverse biological functions, including tumor proliferation, metabolic reprogramming, resistance to cell death, metastasis, immune escape, and cellular autophagy, by targeting specific substrates for ubiquitination. Importantly, TRIM21 exhibits context-dependent effects in cancer, acting either as a tumor suppressor or oncogene, depending on the target. Finally, they discuss the potential of TRIM21 as a therapeutic target and suggest that better understanding of its context-specific functions could inform novel cancer therapies.

Despite substantial advances in E3 ligase–based therapeutic strategies, significant gaps remain in the field. Although more than 600 human E3 ligases have been identified, the majority of current PROTACs rely on a limited subset, most notably CRBN and VHL (Barik et al., 2023; Girardini et al., 2019). Similarly, molecular glues that induce neo-substrate recruitment are known to engage the E3 ligases CRBN and DCAF15 (Hu et al., 2024). Recent efforts have therefore focused on expanding the E3 ligase repertoire for targeted protein degradation, with alternative ligases, including FBXO22, DCAF1, DCAF15, DCAF16, TRIM21, MDM2, KEAP1, and RNF126, being leveraged to induce the selective degradation of cancer-associated proteins (Islam et al., 2025; Belcher et al., 2023). TRIM21 has emerged as a versatile E3 ligase for targeted protein degradation, particularly through clustering-dependent activation mechanisms that can selectively degrade multimeric or aggregated proteins. Notably, TRIM21-based molecular glue degraders, such as acepromazine, promote the degradation of nuclear pore proteins, thereby disrupting nucleocytoplasmic trafficking. The conversion of acepromazine into PROTACs enables the selective degradation of multimeric proteins, including those within biomolecular condensates, with minimal impact on monomeric counterparts (Lu et al., 2024). Consistently, TRIM21-based small-molecule degraders preferentially eliminate protein aggregates and oligomers, supporting a clustering-based activation mechanism that mirrors TRIM21’s physiological role in antibody-mediated Trim-Away (Luptak et al., 2025). Moreover, a TRIM21-based bioPROTAC selectively degrades HuR, an oncogenic RNA-binding protein, and exerts anti-tumorigenic effects in preclinical models (Fletcher et al., 2023).

Collectively, these findings underscore the growing versatility of TPD platforms and highlight a fundamental conceptual shift in drug discovery from transient inhibition of protein activity to protein degradation. PROTACs can overcome several limitations of conventional small molecule inhibitors, such as degrading mutated isoforms and simultaneously targeting both enzymatic and scaffolding functions of proteins by eliminating the entire protein, thus reducing drug resistance generally associated with small molecule inhibitors (Kim et al., 2023; Yang et al., 2020; Khan et al., 2020). More importantly, by leveraging the tissue-selective expression of specific E3 ligases, PROTACs can exhibit tumor- or tissue-selective activity. For example, a VHL-based PROTAC targeting the BCL-xL antiapoptotic protein, dubbed DT2216, significantly reduces platelet toxicity associated with conventional BCL-xL inhibitors because of the minimal expression of VHL E3 ligase in platelets compared to tumor cells (Khan et al., 2019). Looking ahead, the systematic expansion of the E3 ligase toolbox through chemo-proteomics, structure-guided ligand discovery, and high-throughput screening, coupled with a deeper mechanistic understanding of ligase-specific substrate recognition, tissue selectivity, and regulatory context, will be essential for broadening the therapeutic scope of TPD. The emerging modalities, such as molecular glues, covalent degraders, and conditionally activated PROTACs, bioPROTACs, offer opportunities to enhance degradation selectivity, spatiotemporal control, and clinical safety. Integrating these strategies with cancer genomics and proteostasis network analyses may ultimately enable the rational design of next-generation degraders tailored to tumor-specific vulnerabilities, resistance mechanisms, and complex oncogenic protein assemblies.
